# Nonprecious Single Atom Catalyst for Methane Pyrolysis

**DOI:** 10.3390/molecules29194541

**Published:** 2024-09-25

**Authors:** Naomi Helsel, Sanchari Chowdhury, Pabitra Choudhury

**Affiliations:** Chemical Engineering Department, New Mexico Tech, Socorro, NM 87801, USA; naomi.helsel@student.nmt.edu (N.H.); sanchari.chowdhury@nmt.edu (S.C.)

**Keywords:** methane pyrolysis, nickel-based catalyst, DFT, C-H bond activation

## Abstract

The development of a suitable catalytic system for methane pyrolysis reactions requires a detailed investigation of the activation energy of C-H bonds on catalysts, as well as their stability against sintering and coke formation. In this work, both single-metal Ni atoms and small clusters of Ni atoms deposited on titanium nitride (TiN) plasmonic nanoparticles were characterized for the C-H bond activation of a methane pyrolysis reaction using ab initio spin-polarized density functional theory (DFT) calculations. The present work shows the complete reaction pathway, including energy barriers for C-H bond activation and dehydrogenated fragments, during the methane pyrolysis reaction on catalytic systems. Interestingly, the C-H bond activation barriers were low for both Ni single-atom and Ni-clusters, showing the energy barriers of ~1.10 eV and ~0.88 eV, respectively. Additionally, single-atom Ni-TiN showed weaker binding to adsorbates, and a net endothermic reaction pathway indicated that the single-atom Ni-TiN was expected to resist coke formation on its surface. However, these Ni single-atom catalysts can sinter, aggregate into a small cluster, and form a coke layer from the highly exothermic reaction pathway that the cluster takes despite the facile reaction pathway.

## 1. Introduction

Hydrogen is a key component in sustainable energy production, such as fuel cells, and is considered a clean fuel. However, steam reforming and fossil fuel gasification are the main industrial-scale methods of hydrogen production. This leads to the generation of greenhouse gasses (GHGs), which is an unfortunate consequence of these processes. One way to produce hydrogen without GHG emissions is through methane pyrolysis. The thermal decomposition of methane into hydrogen gas and solid carbon allows for the generation of hydrogen gas while avoiding the generation of carbon dioxide, unlike industrially relevant processes. Acting as a bridge between fossil fuels and renewable energy, large-scale methane pyrolysis could allow for a temporary solution that would bridge the gap in current technology. However, to bridge this gap, a cost-effective catalyst that can balance selectivity, activity, and stability is required [1].

Due to the strong C-H bonds present in methane, temperatures above 1000–1200 °C are required to thermally decompose methane without a catalyst [2]. The operating temperature can be significantly decreased through the use of a catalyst. Transition metal catalysts, such as nickel, iron, and cobalt, have been researched extensively due to their partially filled 3d orbitals, which are able to accept the electrons from C-H bonds easily [1,3].

Showing the highest initial activity among the transition metal catalysts, nickel catalysts are among the most promising catalysts for this process [4]. However, nickel catalysts are susceptible to carbon coking and poisoning when above 600 °C and can degrade rapidly [2,5]. Supports have been researched extensively to improve stability and reduce the thermal sintering that occurs in unsupported catalysts [1,2,6,7]. The performance of these nickel-based catalysts is based on balancing metal–support interactions and the reducibility and dispersion of metal particles on the surface [8,9,10].

The reaction pathway required to complete methane pyrolysis has been recently simulated for single atom Pt on a Cu(111) support to follow the molecular adsorption reaction pathway of methane pyrolysis. In Marcinkowski et al.’s work, it was observed that SAA Pt/Cu (111) support was coke-resistant due to its moderate adsorbate binding and net endothermic reaction pathway. These properties were absent in the Pt(111) catalyst that was susceptible to coking [11].

Recently, our group reported the facile light-mediated synthesis of single-atom Ni catalysts deposited on titanium nitride (TiN) plasmonic nanoparticles. It was found that single Ni atoms favorably deposited on N-vacancy sites on the TiN surface [12]. In the present study, the methane pyrolysis reaction pathway of single atom and small cluster Ni catalysts on TiN supports was investigated. The weaker binding of adsorbates and a net endothermic pathway was observed through DFT calculations and is expected to resist coke formation on a single atom surface. The activation energy observed in the single atom case requires over 30% less energy with an activation energy of ~1.10 eV compared to the Pt/Cu(111) catalyst made by Marcinkowski et al., which has an activation energy of 1.64 eV [11]. However, these calculations also show that, due to the energy required, the catalyst may be able to sinter and aggregate into small clusters, and these clusters are computed to be more active than single atoms with an activation energy of just less than 50% of the energy required by the Marcinkowski et al.’s Pt/Cu(111) catalyst [11]. Though beyond the scope of this study, experiments should be conducted to determine the temperature required to facilitate this reaction using this catalyst and to define the degree of sintering and long-term stability of this catalyst to determine its viability.

## 2. Results and Discussion

**Figure 1:**
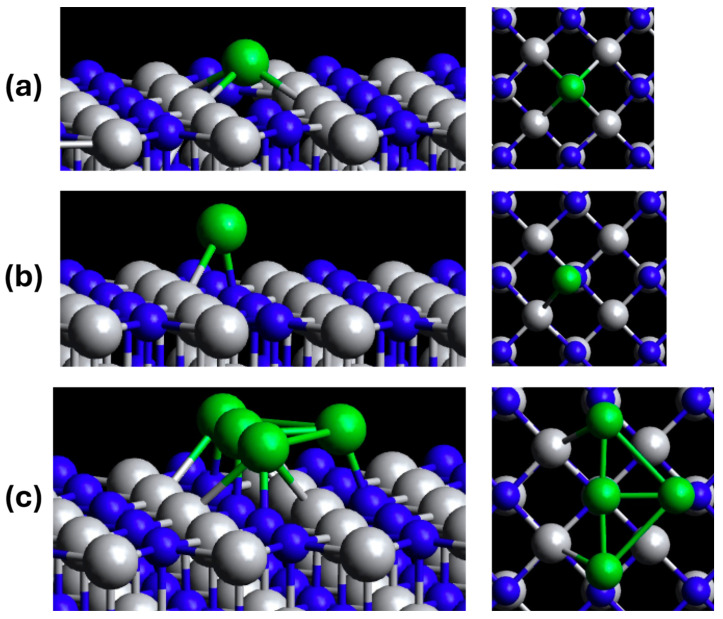
Schematic representations of (**a**) N-vacancy (d-Ni-TiN), (**b**) pristine sites of single-atom Ni-TiN (p-Ni-TiN), and (**c**) 4-atom cluster Ni-TiN (c-Ni-TiN). Green, blue, and silver represent nickel, nitrogen, and titanium, respectively.

Our previous work covers the stable binding sites of titanium nitride (TiN) and titanium dioxide (TiO_2_). This study focuses on the unoxidized surface of TiN instead of its oxidized counterparts of titanium oxy-nitride (TiON) or TiO_2_. It was concluded from previous experimental work that single atoms favor the nitrogen-vacancy sites of TiN and were supported by computational aggregation energy calculations [12]. In Figure 1, schematic representations of the studied Ni-TiN variants are shown.

In the present study, spin-polarized DFT calculations were used to investigate the C-H bond scission steps of methane through to atomic carbon for the defect and N-top TiN sites, which are the two most stable sites of Ni-TiN. The formation energies of C*_x_*H*_y_* are provided in Table S1. The C-H bond scission steps can be characterized by the following reactions:

$${CH}_{4}^{*}\to CH_{3}^{*}+H^{*}\;(TS1)$$

$${CH}_{3}^{*}\to CH_{2}^{*}+H^{*}\;(TS2)$$

$${CH}_{2}^{*}\to CH^{*}+H^{*}\;(TS3)$$

$${CH}^{*}\to C^{*}+H^{*}\;(TS4)$$

**Figure 2:**
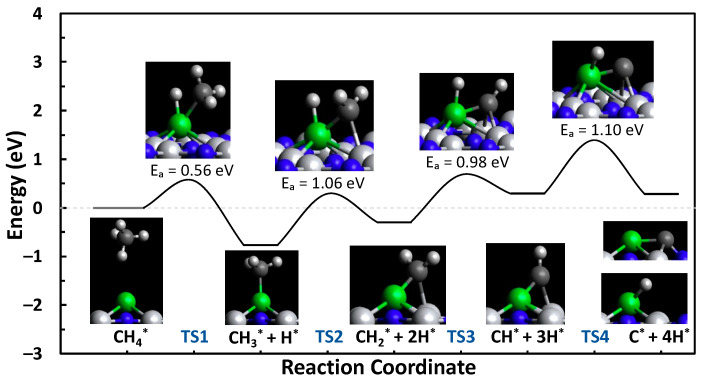
Methane pyrolysis reaction pathway for the defect site of Ni-TiN.

**Figure 3:**
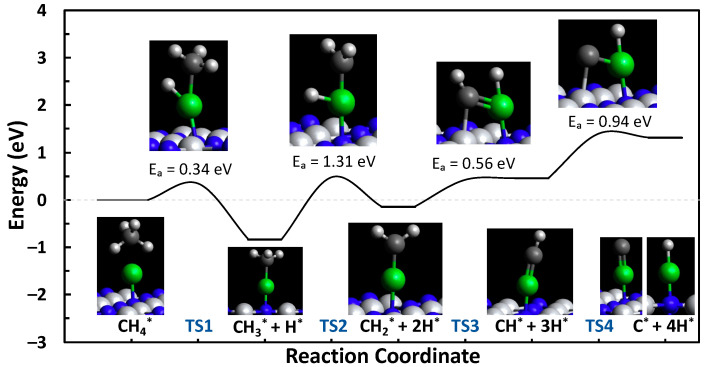
Methane pyrolysis reaction pathway for the N-top site of Ni-TiN.

The formation, activation, and reaction energies of each transition state are provided in Table S2. Using the Dimer method, the transition states of the C-H bond scission steps from methane to atomic carbon were calculated for both the N-vacancy (defect) and N-top (pristine) sites of Ni-TiN. The reaction pathways and the visual representation of the transition states and intermediates are shown in Figure 2 and Figure 3. The defect and N-top sites of Ni-TiN have activation energy barriers of 1.10 and 1.31 eV, respectively. Showing an overall net endothermic reaction pathway and moderate formation energies of the C*_x_*H*_y_* intermediate species, as shown in Table S1, this suggests resistance to coke formation on the surface of both the N-vacancy and N-top active sites of TiN. Moderate formation energies would mean that surface-bound species are not tightly bound to the catalyst surface and would be relatively easy to remove due to their more positive formation energies. It is important to note that the energies in the landscape are in reference to the formation energy of CH_4_ on the surface of the catalyst and that it is more thermodynamically favorable to have CH_4_ adsorbed on the surface over products when the reaction is net-endothermic.

**Figure 4:**
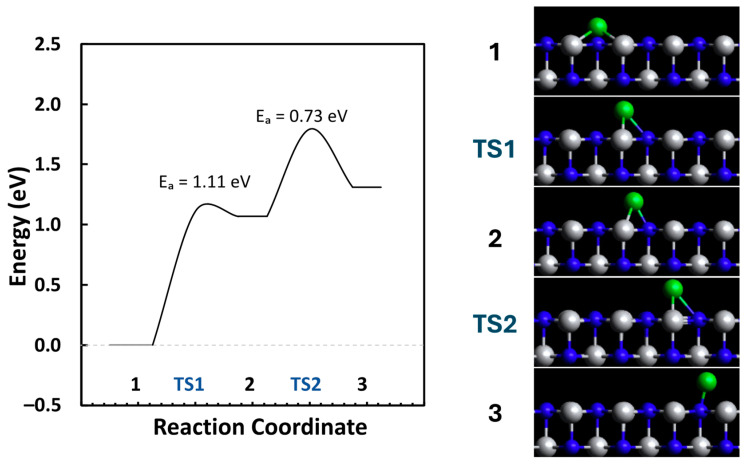
Diffusion landscape for the N-vacancy to the N-top site of Ni-TiN.

As per our previous publication, single atoms seem preferable on the N-vacancy site, but small clusters seem preferable on the N-top site [12]. Because of this, we investigated the possibility of site diffusion from N-vacancy to the N-top site using dimer simulations, as shown in Figure 4. From Figure 4, we can see that there is a stable site in between the N-vacancy to the N-top site that sits at the edge of the defect. This site is less preferential than N-vacancy since its binding energy is only −3.91 eV, and the N-vacancy sites would be filled first (binding energy of −4.98 eV); this means that this site would only be present at the possibility of sintering when adequate energy is provided. If the diffusion energy barrier is equivalent or less than the activation energy barrier of the methane pyrolysis reaction, then sintering would be expected to occur because of the amount of energy provided. Some N-vacancy sites would migrate to the N-top site and form a small cluster because the activation and diffusion barriers are nearly equivalent. In this case, providing the amount of energy necessary for methane pyrolysis would mean that we would need to also provide the amount of energy needed for Ni atoms to diffuse to different sites, and there is no way to control which process is preferred.

**Figure 5:**
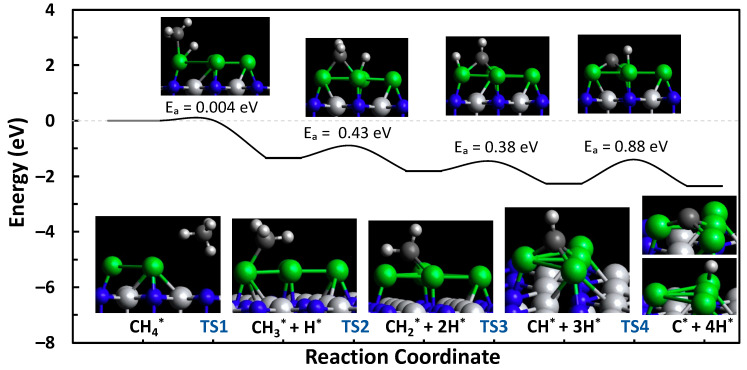
Methane pyrolysis reaction pathway for the 4-atom cluster of Ni-TiN.

We investigated the methane pyrolysis of a small four-atom Ni cluster, as shown in Figure 5 (c-Ni-TiN). A four-atom Ni cluster was chosen because that is where the aggregation energy flattened off for p-Ni-TiN in our previous publication, meaning that the five-atom cluster was not any more favorable than the four-atom [12]. Additionally, it was expected that the Ni-Ni interactions present in the cluster system would be adequate enough to represent a different system, which was later noted in the difference in charge distribution, binding to intermediates, and better overall activation energies, which is indicative of Ni-based catalysts for this process. The activation energy of c-Ni-TiN is interesting, with a near-instantaneous first C-H bond break and a lower barrier of 0.88 eV. Tables S4–S6 show the formation energies of all intermediate binding locations tested and the transition states, in addition to the vibrational frequency confirmations of each of the transition states. When comparing single-atom Ni-systems (p-Ni-TiN and d-Ni-TiN) with the cluster system (c-Ni-TiN), we can see a considerable difference in the formation energies of smaller C_x_H_y_ species like CH and C. These species are easier to form on the surface of the cluster, which is probably why the activation energy is lower, but, in turn, this could also contribute to c-Ni-TiN coking.

**Figure 6:**
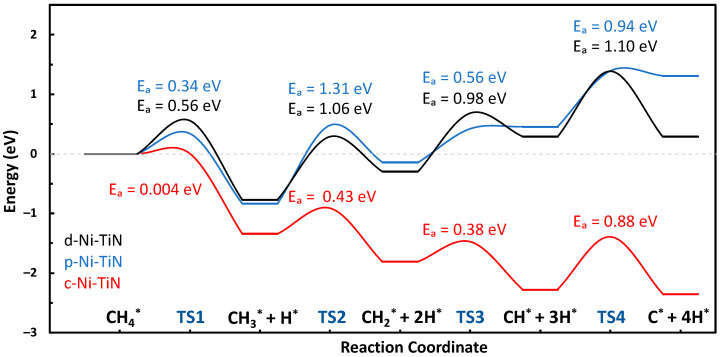
Comparison of methane pyrolysis reaction pathways for d-Ni-TiN, p-Ni-TiN, and c-Ni-TiN.

Figure 6 shows a comparison of the methane pyrolysis reaction pathways for all three variants. The finding of a lower activation energy from the cluster over the single atom is plausible because of nickel’s robust ability to break the C-H bond. In recent studies in the literature, nickel-based catalysts have been shown to have the highest initial activity when compared to other metal-based catalysts [1]. That being said, the reaction landscape shows a highly exothermic reaction pathway, suggesting the formation of coke on the surface. Nickel’s high activity generally comes at a price, and it generally suffers from deactivation due to the imbalance of carbon generation and diffusion when a catalyst is not coke-resistant; simply speaking, the carbon cannot diffuse fast enough after being generated. Experimental research should be conducted on this catalyst whilst observing the sintering that would occur as a result of the pyrolysis.

To avoid the sintering of the single-atom catalyst, any reaction barrier should be significantly less than the diffusion barrier. Different applications (non-oxidative coupling) should be explored for this catalyst. Due to the near-instantaneous first transition state of the c-Ni-TiN system, little energy should need to be provided to facilitate this step, which could allow for other reactions to be favored over methane pyrolysis for subsequent steps based on the amount of energy provided. Another capability of this catalyst that is used during synthesis is titanium nitride’s plasmonic properties. These properties could be used to lower the activation energy barrier of a given reaction. TiN’s plasmonic properties could allow for the generation of hot carriers when exposed to light, which could decrease activation energy due to an increase in stored vibrational bond energy between the catalyst and reaction intermediate [13].

While beyond the scope of this research, future work should be conducted to investigate the use of a co-dopant, such as palladium or copper, which are common promoters of nickel catalysts that do not interact with methane dissociation due to their filled orbitals but can alter nickel’s electronic properties significantly. The introduction of copper to a Ni-based catalyst supported on carbon nanotubes results in an enhanced reaction temperature, methane conversion, and carbon yields [14]. Though not explicitly stated, it is expected that Cu alters the properties of the Ni-based catalyst favorably even when Cu itself does not generally have a high activity towards this process. The introduction of palladium was recently investigated for a C-C coupling reaction using a Ni-Pd catalyst in which they found that there was significant Ni-Pd charge transfer that resulted in a highly negatively charged Pd center [15]. These property-changing co-dopants could reduce the amount of sintering that occurs due to increased stability and potentially decreased active site diffusion [1,14,15].

**Figure 7:**
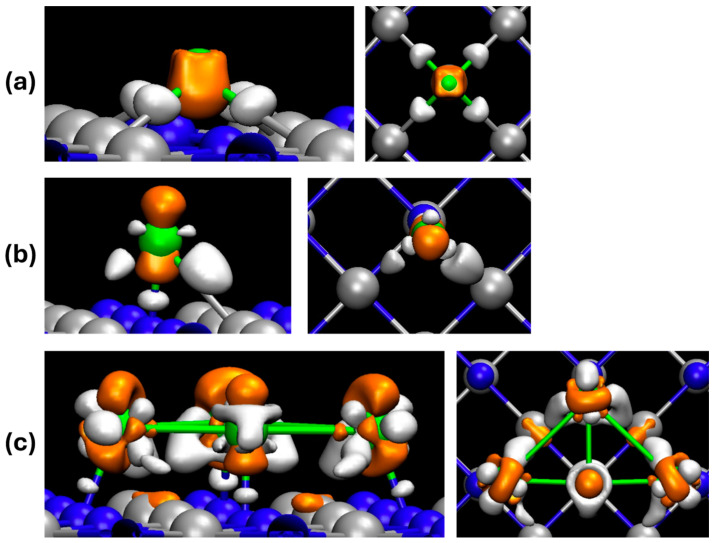
Charge density differences in Ni deposited on TiN for (**a**) d-Ni-TiN, (**b**) p-Ni-TiN, and (**c**) c-Ni-TiN. Orange represents the accumulation of charge, while white represents depletion. An iso-surface value of ±0.07 e^−^/Å^3^ was used for each case.

To obtain a deeper understanding qualitatively of why the small Ni cluster catalyst performs better than the single atom catalyst and why the diffusion energy barrier of single atom Ni is so low, we opted to look into the electronic properties using charge density differences (CDDs), and partial density of state (PDOS) plots. The CDD plots shown in Figure 7 allow us to visualize where a charge is accumulated and depleted in the catalyst. In each system, the Ni atoms have different interactions with the TiN substrate. In the d-Ni-TiN system, Ni is interacting with the neighboring Ti atoms, which is causes a depletion zone between the Ni and Ti atoms. In the p-Ni-TiN system, the Ni is interacting unevenly with both Ti and N atoms while also showing less charge interactions between Ni and the substrate. The c-Ni-TiN system shows the highest interaction with the substrate, and the four Ni atoms are mainly interacting with the Ni atoms in the TiN substrate. This shows that more charge accumulation is being drawn towards the N atoms and is leaving larger depletion zones around the Ni atoms. It appears that c-Ni-TiN more partially fills Ni states and has more opportunity to fill electrons versus single-atom systems. Additionally, d-Ni-TiN has more depletion zones than p-Ni-TiN, which means it has more opportunity to accept a charge from intermediates. Due to the number of accumulation and depletion zones in the same iso-surface value, it is probable that the cluster is more active than the single atom, which results in a lower activation energy.

We also calculated the partial density of state plots for each of the transition states for d-Ni-TiN, p-Ni-TiN, and c-Ni-TiN, as shown in Figures S1–S3 in the Supplementary Materials. In the case of the cluster Ni-TiN, the orbitals/electrons of all four Ni atoms were considered in the PDOS plot for simplicity. These PDOS plots allowed for the confirmation of chemical interactions between the Ni and C atoms from the transition state. This was achieved by looking at the valence orbitals of both the C and Ni atom(s) and looking for overlap. Each figure shows at least some overlap between a C *p* orbital and a Ni *d* orbital. This confirms weak/moderate bonding between the nickel atom and the C atom from the transition state.

The d-band centers of the Ni atoms for each initial and intermediate system were plotted against activation energy and carbon formation energy, as seen in Figures S4 and S5 in the Supplementary Information. This was conducted to see if there was any immediate correlation between the Ni d-band center and activity or carbon formation. No clear trend was drawn from these plots.

**Figure 8:**
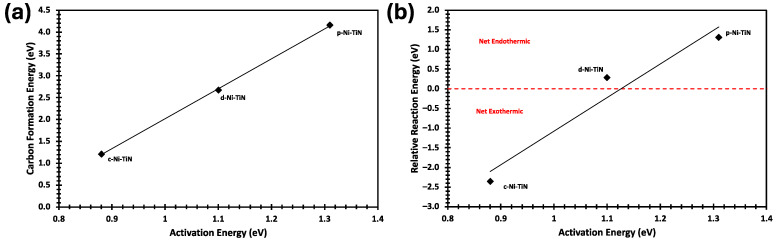
(**a**) Carbon formation energy and (**b**) relative reaction energy were both studied against the highest activation energy barriers for each of the Ni system variants (d-Ni-TiN, p-Ni-TiN, and c-Ni-TiN).

To further investigate why d-Ni-TiN is an advantageous catalyst to study, carbon formation energy and relative reaction energy are plotted against activation energy (the highest TS barrier for each variant) in Figure 8. A directly proportional trend is found in both plots from Figure 8a,b, which indicates that the more active the nickel catalyst is, the easier it is to form carbon on the surface. It can also be noted that the trend lines increase with both the overall exothermic and endothermic reaction pathways. The results also suggest that d-Ni-TiN is the better choice for a catalytic system among all the three systems studied. Being essentially a midpoint, d-Ni-TiN has a moderate activation energy and carbon formation energy while still being a net endothermic reaction pathway, which is not expected to form coke.

## 3. Computational Methods

Utilizing Vienna Ab Initio Simulation Package (VASP) version 5.4.1, spin-polarized density functional theory calculations were carried out [16,17,18]. The Perdew–Burke–Ernzerhof (PBE) form of the generalized gradient approximation (GGA) was used to describe the interactions between valence electrons and frozen cores [19]. A semi-empirical scheme proposed by Grimme (DFT-D2) was used to account for Van der Waal interactions. The projected-augmented wave method (PAW) was used with a 400 eV energy cut-off [20,21]. With a width of 0.05 eV around the Fermi level, Gaussian smearing was used to facilitate convergence. Electronic energies converged to 10^−6^ eV, and ionic relaxations were performed until residual forces on the atom were less than 0.02 eV/Å. The Brillouin zone was sampled using a 3 × 3 × 1 Monkhorst-Pack *k*-point mesh for all relaxation simulations, while density of state (DOS) calculations utilized a 9 × 9 × 1 Monkhorst-Pack k-point mesh.

The cell was periodic in the x/y direction and was 25 Å in the z direction to ensure negligible interactions between periodic images. The pristine titanium nitride (TiN) cell was 14.97 × 14.97 Å in the x and y directions, comprising 50 titanium atoms and 50 nitrogen atoms. The defective TiN cell comprised the same dimensions and titanium atoms but had 1 less nitrogen atom.

The bond scission steps of the reaction pathway were determined using the Dimer method [22,23], utilizing the conjugate gradient optimizer, and were confirmed with vibrational frequency analysis. The formation energies were calculated using the formula used by Marcinkowski et al. and is shown below for a given C*_x_*H*_y_* species (where x=0,1 and y=0,1,2,3,4) [11]:

$$E_{f}\left(C_{x}H_{y}\right)=E_{tot}\left(C_{x}H_{y}+slab\right)-\left\{E_{tot}\left(slab\right)+x\cdot E_{tot}\left(CH_{4}\right)-\left(\frac{4x-y}{2}\right)\cdot E_{tot}\left(H_{2}\right)\right\}$$

The formation energies are given with respect to gas-phase CH_4_ and H_2_. In this equation, the DFT total energy of the C*_x_*H*_y_* intermediate, bound to the catalyst surface (*E_tot_*(C*_x_*H*_y_ +* slab)), is subtracted by the sum of the DFT total energies of the catalyst surface (*E_tot_*(slab)) and gas-phase methane reactant (*E_tot_*(CH_4_)) or hydrogen product (*E_tot_*(H_2_)) multiplied by the subscript *x* or *y* for CH_4_ and H_2_ depending on which intermediate is being formed. This equation, which shows the formation of energies of surface-bound species, is given with respect to gas-phases CH_4_ and H_2_, which have formation energies of zero when taken as a reference. The activation energy is written as the difference between the DFT transition state total energy ($E_{tot}^{‡}$) and DFT initial state total energy ($E_{tot}^{IS}$).

$$E_{a}=E_{tot}^{‡}-E_{tot}^{IS}$$

The charge density differences (CDDs) of the initial systems were calculated using the following equation:

$$\Delta \rho =\rho_{Ni-TiN}-\rho_{TiN}-\rho_{Ni}$$

where *ρ_Ni-TiN_*, *ρ_TiN_*, and *ρ_Ni_* are the charge density of the combined system, the isolated TiN substrate, and the Ni atom or atoms, respectively. The latter two charge densities utilize a fixed geometry at the optimized geometry of the combined system.

## 4. Conclusions

As a source of sustainable hydrogen produced from natural gas, methane pyrolysis could be a temporary solution to bridge the gap between fossil fuels and renewable energy. The current catalysts used for this process deactivate easily due to coke formation and thermal sintering. This has been reported to be caused by the strong binding of CH*_x_* intermediates and a net exothermic reaction pathway; as such, coke formation is the crux to nickel-based catalysts for methane pyrolysis. In this work, a single-atom nickel catalyst supported by titanium nitride was investigated for use with methane pyrolysis. The present work shows the simulation of the methane pyrolysis reaction pathway utilizing ab initio spin-polarized DFT calculations to elucidate the energy barriers of the single-atom catalyst. Upon investigation, it was found that the single-atom catalyst was expected to be coke-resistant, and the activation energy required to facilitate methane pyrolysis, though considerably low for this process, was similar to the diffusion barrier of the nickel atoms that allow for potential sintering to occur. This could lead to the clustering of nickel atoms into small clusters. A small cluster was then also investigated and was found to have a more favorable activation energy than that of the single atom. However, it has the potential to form a coke layer due to an exothermic reaction pathway. This coke formation could be prevented via the co-doping of other metals, such as palladium or copper, which can have indirect effects on carbon formation energy, activation energy, and relative reaction energy due to their direct effects on the electronic properties of nickel. This needs further investigation, which is beyond the scope of this work.

In all, this work suggests that the single-atom Ni-TiN variant is expected to resist coke formation on its surface but can sinter, aggregate into a small cluster, and form a coke layer from the highly exothermic pathway that this cluster takes. A clear linear trend was found in the studied Ni-TiN variants, which suggest that d-Ni-TiN is a happy medium between the three studied variants in terms of activation energy and carbon formation energy while still being a net endothermic reaction and not being expected to form coke on its surface. It is also recommended that co-dopants, such as copper or palladium, be used to alter the electronic properties, increase stability, and decrease active site diffusion to avoid sintering for this catalyst.

## Data Availability

The original contributions presented in the study are included in the article/Supplementary Material, further inquiries can be directed to the corresponding author.

## Supplementary Materials

The following supporting information can be downloaded at https://www.mdpi.com/article/10.3390/molecules29194541/s1. Figure S1: Partial Density of States of the Ni d orbitals and C p orbitals for (a) TS1, (b) TS2, (c) TS3 and (d) TS4 of d-Ni-TiN; Figure S2: Partial Density of States of the Ni d orbitals and C p orbitals for (a) TS1, (b) TS2, (c) TS3 and (d) TS4 of p-Ni-TiN; Figure S3: Partial Density of States of the Ni d orbitals and C p orbitals for (a) TS1, (b) TS2, (c) TS3 and (d) TS4 of c-Ni-TiN; Figure S4: Initial Ni d-band center plotted as a function of (a) activation energy and (b) carbon formation energy; Figure S5: Ni d-band center plotted as a function of activation energy formatted as series against (a) transition states and (b) Ni system variants (pristine, cluster, defect); Table S1: Formation energies of C*_x_*H*_y_* for both the N-vacancy and N-top sites of Ni-TiN; Table S2: Formation, activation, and reaction energies of each transition state for both the N-vacancy and N-top sites of Ni-TiN; Table S3: Finite differences method vibrational frequencies of each transition state for both the N-vacancy and N-top sites of Ni-TiN; Table S4: Formation energies of all stable C*_x_*H*_y_* for c-Ni-TiN; Table S5: Formation, activation, and reaction energies of each transition state for c-Ni-TiN; Table S6: Finite differences method vibrational frequencies of each transition state for c-Ni-TiN.
