# Supplementary material for: Nonprecious Single Atom Catalyst for Methane Pyrolysis
Source: Molecules. 2024 Sep 25;29(19):4541. doi: 10.3390/molecules29194541 (PMC11477935; doi:10.3390/molecules29194541)
Supplement: Supplementary file 1 [file molecules-29-04541-s001.zip › molecules-3186688-supplementary.pdf]

Supporting Information: Nonprecious Single Atom Catalyst for Methane Pyrolysis

Naomi Helsel, Sanchari Chowdhury, and Pabitra Choudhury*
Chemical Engineering Department, New Mexico Tech, Socorro, NM 87801 USA
*Corresponding Author's E-mail: pabitra.choudhury@nmt.edu

Table S1. Formation energies of C_xH_y for both the N-vacancy and N-top sites of Ni-TiN.

Ni-TiN Site	Species	Formation Energy (eV)	DFT Energy (eV)
N-vacancy	CH_4	-0.09	-969.516
	CH_3	-0.24	-966.277
	CH_2	0.85	-961.800
	CH	2.05	-957.203
	C	2.67	-953.196
	H	-0.62	-949.385
	Bare	n/a	-945.378
N-top	CH_4	-0.65	-980.432
	CH_3	-0.61	-977.001
	CH_2	0.96	-972.044
	CH	2.43	-967.180
	C	4.16	-962.059
	H	-0.88	-959.997
	Bare	n/a	-955.731

Table S2. Formation, activation, and reaction energies of each transition state for both the N-vacancy and N-top sites of Ni-TiN.

Ni-TiN Site	Reaction	E_f(TS) (eV)	E_a (eV)	ΔE_{rxn} (eV)	Dimer Energy (eV)
N-vacancy	TS1	0.47	0.56	-0.77	-968.961
	TS2	0.82	1.06	0.47	-965.222
	TS3	1.83	0.98	0.59	-960.819
	TS4	3.16	1.10	0.0003	-956.102
N-top	TS1	-0.31	0.34	-0.83	-980.091
	TS2	0.70	1.31	0.69	-975.688
	TS3	1.52	0.56	0.60	-971.482
	TS4	3.37	0.94	0.86	-966.243

Table S3. Finite differences method vibrational frequencies of each transition state for both the N-vacancy and N-top sites of Ni-TiN.

**Reaction**	**Vibrational Frequencies (cm^{-1})**																	
	**V_1**	**V_2**	**V_3**	**V_4**	**V_5**	**V_6**	**V_7**	**V_8**	**V_9**	**V_{10}**	**V_{11}**	**V_{12}**	**V_{13}**	**V_{14}**	**V_{15}**	**V_{16}**	**V_{17}**	**V_{18}**
N-vacancy																		
TS1	3029	2965	2742	1971	1437	1356	1185	832	740	435	315	168	149	125	118	82	71	954i
TS2	3018	2948	1895	1315	877	800	548	465	353	323	178	145	121	111	700i			
TS3	3030	1889	842	678	519	368	356	232	183	150	106	578i						
TS4	1831	698	440	399	296	188	146	89	773i									
N-top																		
TS1	3041	3005	2889	2137	1400	1375	1178	840	749	488	277	242	151	76	49	32	22i	717i
TS2	3016	2938	1666	1323	851	742	642	495	261	237	177	136	80	41	448i			
TS3	3027	2007	988	715	580	403	335	262	151	147	67	469i						
TS4	3058	871	574	443	282	143	137	60	108i									

Table S4. Formation energies of all stable C_xH_y for c-Ni-TiN.

**Ni-TiN Site**	**Species**	**Site**	**Formation Energy (eV)**	**DFT Energy (eV)**
c-Ni-TiN	CH_4	1	-0.17	-994.384
		2	-0.15	-994.462
		3	-0.08	-994.446
	CH_3	1	-0.57	-990.794
		2	-0.26	-991.170
		3	0.11	-991.476
	CH_2	1	-0.11	-991.477
		2	0.13	-987.385
	CH	1	0.36	-987.625
	C	1	1.21	-987.385
		2	2.41	-987.385
		3	2.45	-983.768
	H	1	-0.93	-983.768
		2	-0.80	-983.768
	Bare	n/a	n/a	-970.245

Table S5. Formation, activation, and reaction energies of each transition state for c-Ni-TiN.

Ni-TiN Site	Reaction	E_f(TS) (eV)	E_a (eV)	ΔE_{rxn} (eV)	Dimer Energy (eV)
c-Ni-TiN	TS1	-0.16	0.004	-1.34	-994.458
	TS2	-0.14	0.43	-0.47	-991.046
	TS3	0.23	0.33	-0.47	-987.288
	TS4	1.24	0.88	-0.007	-982.885

Table S6. Finite differences method vibrational frequencies of each transition state for c-Ni-TiN.

Reaction	Vibrational Frequencies (cm^{-1})														
	V_1	V_2	V_3	V_4	V_5	V_6	V_7	V_8	V_9	V_{10}	V_{11}	V_{12}	V_{13}	V_{14}	V_{15}
c-Ni-TiN															
TS1	3033	3028	2926	1790	1397	1357	1155	792	738	422	297	132	100	75	802i
TS2	3007	2946	1963	1332	926	817	586	577	370	333	111	765i			
TS3	3015	1929	976	647	588	456	389	296	541i						
TS4	1859	650	561	423	380	797i									

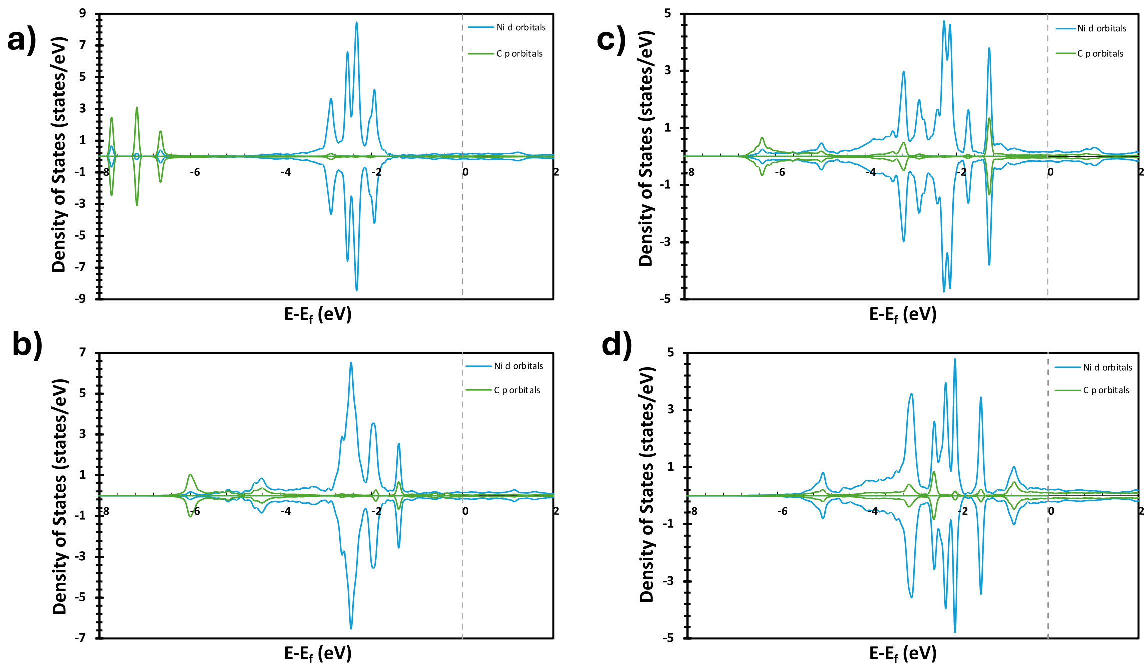

Figure S1. Partial Density of States of the Ni d orbitals and C p orbitals for a) TS1, b) TS2, c) TS3 and d) TS4 of d-Ni-TiN.

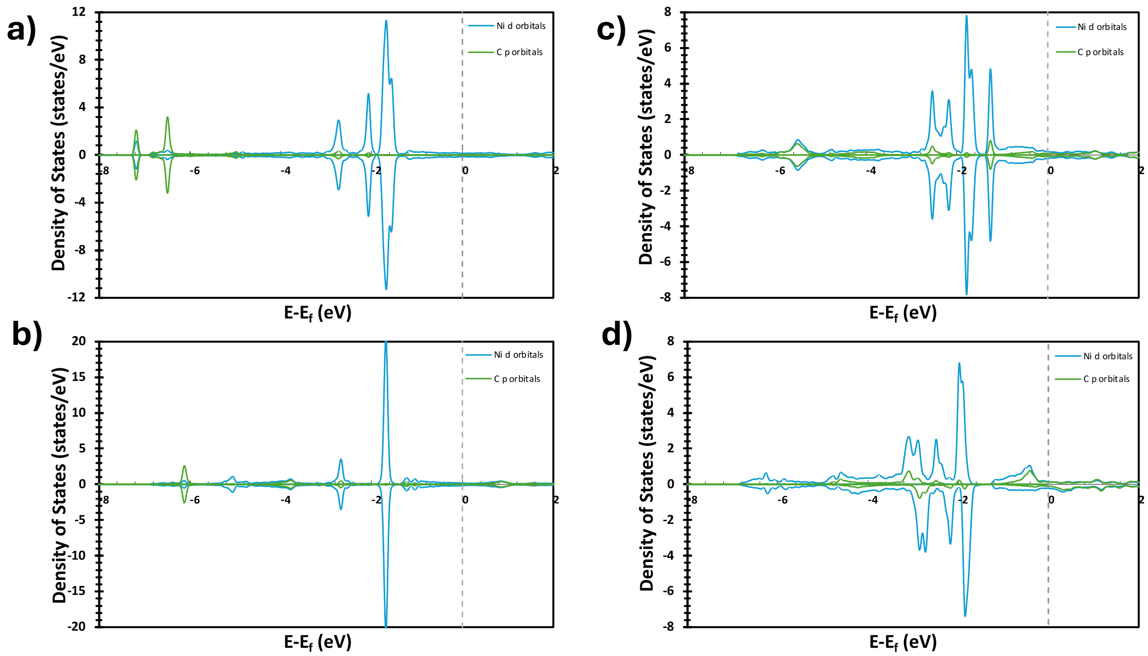

Figure S2. Partial Density of States of the Ni d orbitals and C p orbitals for a) TS1, b) TS2, c) TS3 and d) TS4 of p-Ni-TiN.

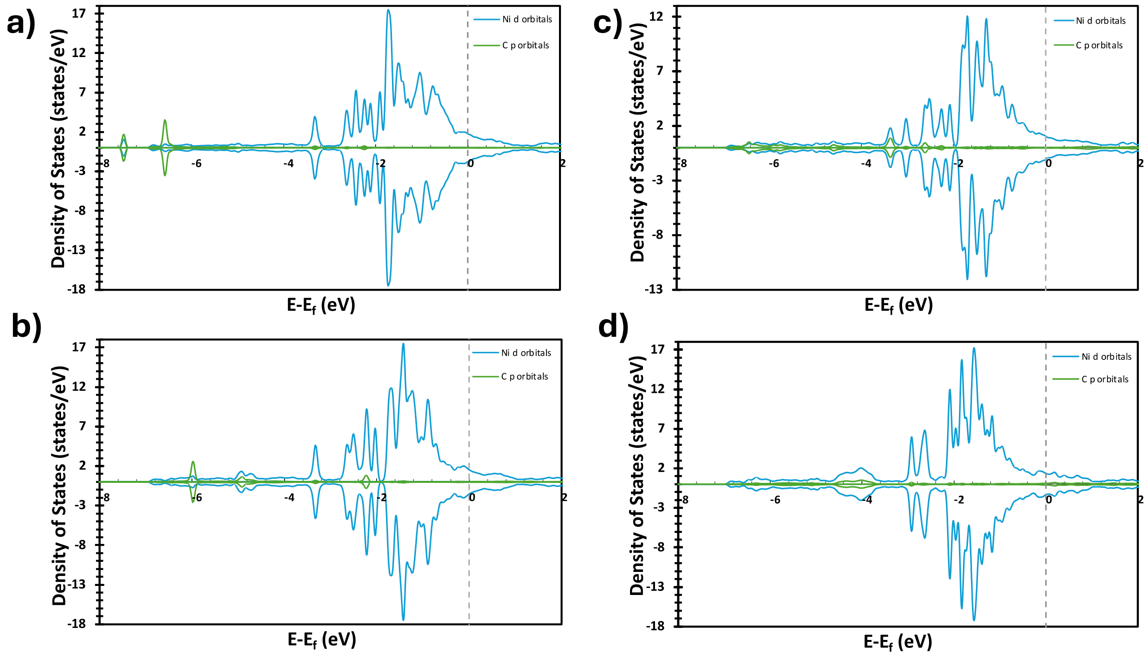

Figure S3. Partial Density of States of the Ni d orbitals and C p orbitals for a) TS1, b) TS2, c) TS3 and d) TS4 of c-Ni-TiN.

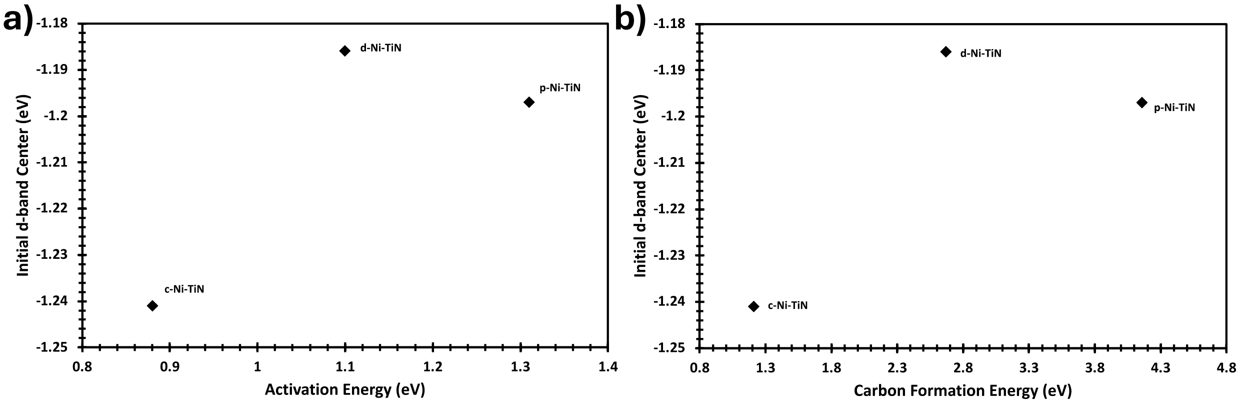

Figure S4. Initial Ni d-band center plotted as a function of a) activation energy and b) carbon formation energy.

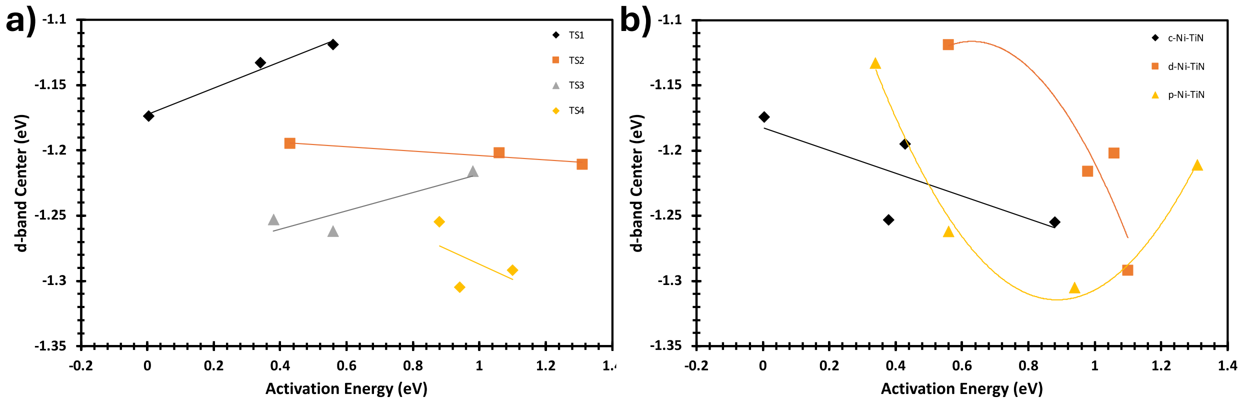

Figure S5. Ni d-band center plotted as a function of activation energy formatted as series against a) transition states and b) Ni system variants (pristine, cluster, defect).
